# Epo Receptors Are Not Detectable in Primary Human Tumor Tissue Samples

**DOI:** 10.1371/journal.pone.0068083

**Published:** 2013-07-04

**Authors:** Steve Elliott, Susan Swift, Leigh Busse, Sheila Scully, Gwyneth Van, John Rossi, Carol Johnson

**Affiliations:** Amgen Inc, Thousand Oaks, California, United States of America; Faculty of Medicine, University of Porto, Portugal

## Abstract

Erythropoietin (Epo) is a cytokine that binds and activates an Epo receptor (EpoR) expressed on the surface of erythroid progenitor cells to promote erythropoiesis. While early studies suggested EpoR transcripts were expressed exclusively in the erythroid compartment, low-level EpoR transcripts were detected in nonhematopoietic tissues and tumor cell lines using sensitive RT-PCR methods. However due to the widespread use of nonspecific anti-EpoR antibodies there are conflicting data on EpoR protein expression. In tumor cell lines and normal human tissues examined with a specific and sensitive monoclonal antibody to human EpoR (A82), little/no EpoR protein was detected and it was not functional. In contrast, EpoR protein was reportedly detectable in a breast tumor cell line (MCF-7) and breast cancer tissues with an anti-EpoR polyclonal antibody (M-20), and functional responses to rHuEpo were reported with MCF-7 cells. In another study, a functional response was reported with the lung tumor cell line (NCI-H838) at physiological levels of rHuEpo. However, the specificity of M-20 is in question and the absence of appropriate negative controls raise questions about possible false-positive effects. Here we show that with A82, no EpoR protein was detectable in normal human and matching cancer tissues from breast, lung, colon, ovary and skin with little/no EpoR in MCF-7 and most other breast and lung tumor cell lines. We show further that M-20 provides false positive staining with tissues and it binds to a non-EpoR protein that migrates at the same size as EpoR with MCF-7 lysates. EpoR protein was detectable with NCI-H838 cells, but no rHuEpo-induced phosphorylation of AKT, STAT3, pS6RP or STAT5 was observed suggesting the EpoR was not functional. Taken together these results raise questions about the hypothesis that most tumors express high levels of functional EpoR protein.

## Introduction

Erythropoietin (Epo) is a late acting growth factor that stimulates red blood cell formation (erythropoiesis) [1] through binding and activating an Epo receptor (EpoR) on the surface of committed erythroid progenitor cells resulting in their survival, proliferation and differentiation. Cloning of the Epo gene in the early 1980s allowed the development of erythropoiesis stimulating agents (ESAs) including recombinant human Epo (rHuEpo) as a treatment for anemia in multiple settings, offering an alternative to transfusion as a method of raising or maintaining hemoglobin levels in patients.

Early reports suggested that response of ESAs was limited to the erythroid compartment due to the restricted expression of EpoR transcripts to erythroid progenitor cells [1]. However, with the introduction of more sensitive RT-PCR strategies, low levels of EpoR transcripts relative to that in erythroid cells were also detected in other tissues and cell types [2]. This raised the possibility that recombinant human Epo (rHuEpo) may have non-erythroid effects [3], [4]. The detection of EpoR transcripts in tumor cells lines led to suggestions that rHuEpo may also act as a tumor growth factor and in turn promote tumor progression. However the EpoR transcript levels were significantly below that found in positive controls (cells or tissues containing Epo-responsive cell types) with no elevation in tumor compared to nontumor tissues [5]. In addition, the EpoR gene itself was only rarely amplified in tumors [5].This suggested that EpoR gene amplification or overexpression of the gene was not a generalized property of tumors. However it was still possible that this low level of EpoR mRNA was translated into significant levels of EpoR protein that was functional and therefore responsive to ESAs.Therefore it was essential to determine if EpoR protein was present.

Investigations of EpoR protein expression in normal and tumors tissues were initially evaluated by immunohistochemistry (IHC) or western blot using anti-EpoR antibodies and positive results were reported [3]. However the antibodies employed were subsequently shown to be non-specific [6]–[8] raising questions about those results. In other studies the anti-EpoR polyclonal antibody M-20, which is a polyclonal antibody raised to a murine EpoR peptide and thought to show some specificity to human EpoR, was used to examine EpoR protein expression in breast cancer samples. According to IHC and western data, breast tumor sections and the breast tumor cell line MCF-7 were reported to express high levels of EpoR protein [6], [9]. However MCF-7 cells were reported elsewhere to express little EpoR protein and be nonresponsive to rHuEpo [8], [10]. Further the specificity of M-20 is in question because M-20 antibodies stained mouse wild type and EpoR knockout tissues similarly by IHC [6].

In the absence of definitive antibody reagents to detect EpoR protein, in vitro experiments were designed with the goal of detecting functional responses with tumor cell types following rHuEpo addition. With the limited availability of live primary cells from tumor biopsies or resections, experiments on tumor cell lines were performed instead. The significance of positive results with cell lines as opposed to primary tumor cells are uncertain and in any case the results reported were conflicting [2]. Further, the positive results reported were inconclusive because of the absence of negative controls which would allow detection of false-positive effects. Such false-positive effects, can occur with trivial manipulations such as a change in growth medium [11], the presence of growth promoting agents in the vehicle [11]–[14] or because of contaminants such as endotoxin in nonsterile preparations [15], [16]. Indeed, most reports of ESA-induced signaling responses with tumor cell lines reported changes in levels of pERK or pAKT, molecules that are phosphorylated by multiple ligand receptor complexes and sensitive to experimental manipulations [17]. Phosphorylation of JAK2 or STAT5 in tumor cell lines with rHuEpo, molecules more specific to the EpoR signaling pathway, was rarely reported. Indeed in several studies there was a notable absence in changes in JAK2 or STAT5 phosphorylation after rHuEpo addition which was in contrast to the positive effects on ERK or AKT phosphorylation in the same studies[18]–[21] further questioning the significance of the existing data. However there was a report of a lung cancer cell line (NCI-H838) that with ESA addition showed increased phosphorylation of STAT5 [22] which is immediately downstream of JAK2 in the signal transduction pathway.

Recently a specific and sensitive antibody to human EpoR was described (A82) and appropriate positive and negative control cell types were identified [23]. Previous results with these reagents demonstrated that nonhematopoietic tissues and cell types expressed little EpoR protein [24], [25]. A western survey with A82 of 66 different tumor cell lines demonstrated that EpoR protein was at no/low levels in most tumor cell lines and that the lines with the highest levels did not signal with ESAs [10].

Here we extend those studies and show that according to westerns using A82, EpoR protein is undetectable in matched normal and cancerous tissues and was low/undetectable in a panel of breast tumor cell lines. Higher, but still relatively low, levels were observed in some lung tumor cell lines including NCI-H838, which was reported elsewhere to signal through STAT5 with rHuEpo [22]. However, reported here and under controlled conditions, NCI-H838, rHuEpo did not stimulate phosphorylation of AKT or STAT5. We also report here that the anti-EpoR antibody M-20 from Santa Cruz Inc, which reportedly detected EpoR protein on breast cancer cell lines, did not detect an EpoR band in human breast tumors but did give false-positive staining by IHC of positive and negative control mouse tissues where the human EpoR gene replaced the murine EpoR. M-20 also bound to a non-EpoR protein similar in size to that of authentic EpoR in some cell lines indicating that western data with M-20 can also be particularly difficult to interpret. Taken together these results raise questions about the hypothesis that most tumors express high levels of functional EpoR protein and that rHuEpo directly stimulates growth/survival of tumor cells.

## Materials and Methods

### Animals, Cells, Cell Lines and Cell Culture

Cell lines were obtained from a variety of sources including American Type Tissue Collection (Manassas, VA); European Collection of Cell Cultures (Salisbury, UK); and German Collection of Microorganisms and Cell Culture (Braunschweig, Germany). EpoR positive control cell lines UT-7/Epo [26] and OCIM-1 [27], [28] were generous gifts from Dr N.Komatsu (Jichi Medical School, Minamikawachi, Japan) and Dr V. Broudy (University of Washington, Seattle) respectively. Cell lines were grown in media as recommended or as described previously [10], [25]. Positive control human erythroid cells were generated from peripheral blood CD34^+^ cells (Stemcell Technologies) by culturing for 4–6 days in StemSpan Serum Free media (Stemcell Technologies) containing 50 ng/ml SCF (R&D Systems, Minneapolis, MN), 10 ng/ml IL-3 (R&D Systems), 10 ng/ml IL-6 (R&D Systems) and 5 U/ml rHuEpo (Amgen). The resulting cells were primarily erythroid progenitors as characterized and described previously [24].

The animals used in this study were cared for in accordance to the Guide for the Care and Use of Laboratory Animals, 8th Edition. All research protocols were approved by the Amgen Thousand Oaks Institutional Animal Care and Use Committee (IACUC); no regulated species were used. Euthanasia was conducted by IAUCU-approved methods and all efforts were made to minimize suffering.

### Preparation of Mouse and Human Tissues

Human tissue samples were from Zoion (Zoion Diagnostics, Hawthorne, NY) and the National Resource Center (Bethesda,MD). Tissues, primary cells and cell lines were collected and prepared for westerns as described previously [24]. Cell preparations examined in western blots were prepared by adding 1X reducing agent (Invitrogen, Carlsbad, CA) and 1X LDS buffer (Invitrogen) and boiling the sample for 10 minutes. Wild type (WT) (C57/Bl6) or homozygous Human *EPOR* knock-in (KI) mice [29] (kindly provided by J. Prchal; University of Utah, Salt Lake City, UT) were injected subcutaneously with either carrier (0.1% BSA in PBS) or with 1 mg/kg (0.2 mL) recombinant mouse Epo (rMsEpo, Amgen) to induce an erythroid response. On day 4 following injection, animals were sacrificed and tissues were collected and either snap-frozen in liquid nitrogen or fixed in 10% neutral-buffered formalin for 24 hours and processed on a Tissue-Tek VIP tissue processor (Sakura Finetek USA, Torrance, CA) into paraffin.

### EpoR Western Immunoblotting

EpoR western blots were prepared and probed with a rabbit monoclonal antibody specific to human EpoR (A82) [23], a rabbit polyclonal antibody raised to the amino terminal extracellular domain of murine EpoR (Amgen Inc), commercially available anti-EpoR polyclonal antibodies (M-20 and C-20 (Santa Cruz Inc; Santa Cruz, CA), ab10653 (Abcam; Cambridge, MA) or a commercially available monoclonal antibody Mab307 (R&D systems; Minneapolis, MN). Anti Her2 antibody was from Cell Signaling Technology. Western immunoblotting was as described previously [10], [23]. Protein concentrations were determined by Bradford assay and equal protein amounts (15 ug) were subjected to SDS-PAGE with the same amount loaded for all lanes of the gel. For loading controls, the membranes were stripped and probed with anti-cyclophilin B, (ab106045; Abcam, Cambridge, MA) or anti-GAPDH (Chemicon). Primary antibodies were used at 0.1 ug/ml (A82), 0.2 ug/ml (M-20), 0.25 ug/ml (Cyclophilin B), 1∶1000 (Her2) and 1∶1000 (GAPDH). Secondary antibodies were Horseradish Peroxidase-linked anti-rabbit IgGs (HRP-IgG; Jackson Laboratories, Bar Harbor, ME) used at 10 ng/ml.

EpoR protein levels were estimated using semi-quantitative western blots by comparing band intensity of purified EpoR-extracellular domain (EpoR-ECD) to the 59 kDa full-length protein in the cell lysate of interest as described previously [10]. In some experiments cell lysates were first immunoprecipitated and the material was then subjected to immunoblotting as described previously [6].

### EpoR Immunohistochemistry (IHC)

Tissue sections (5 µm) were deparaffinized and hydrated in deionized water and pretreated with DIVA Decloaker-antigen retrieval reagent (Biocare # DV200; Concord, CA) in the Biocare Decloaking Chamber (pressure cooker) as recommended by the manufacturer (Biocare # DV200; Concord, CA ). Sections were blocked with CAS BLOCK (Invitrogen, # 00-8120; Camarillo, CA) and incubated for 1 hour at room temperature with 4 µg/ml of A82 monoclonal rabbit antibody (Amgen; Thousand Oaks, CA). Sections were labeled with biotinylated goat anti-rabbit IgG (Vector # BA-1000; Burlingame, CA) at 1∶200 for 25 min and followed with ABC (Vector #PK-6100). Sections were treated with Tyramide Signal Amplification (Perkin Elmer, Inc., # SAT-70000; Waltham, MA) at 1∶200 for 10 minutes and followed with ABC (Vector) again. The reaction sites were visualized with diaminobenzadine (DAB) (DAKO #K3468; Carpinteria, CA) and counterstained with hematoxylin.

For M-20 IHC, dehydrated and deparaffinized sections were blocked with CAS BLOCK (Invitrogen, # 00-8120; Camarillo, CA) and incubated for 1 hour at room temperature with the polyclonal anti-EpoR M-20 (Santa Cruz sc-697, lot #E2004; Santa Cruz, CA) at 1∶200 dilution. Sections were labeled with EnVision-HRP labeled polymer anti-rabbit (DAKO # K4003; Carpinteria, CA). The reaction sites were visualized with DAB (DAKO #K3468; Carpinteria, CA) and counterstained with hematoxylin.

### NCI-H838 Cell Signaling Studies

In order to maximize sensitivity to rHuEpo or growth factor addition, logarithmically growing -NCI-H838 cells were serum starved in basal media +0.1%FBS overnight. The starved cells were then incubated with 1 U/ml or 10 U/ml rHuEpo (Amgen) or an EGF/HGF/IGF-1 cocktail [EGF (100 ng/ml; Roche), HGF (500 ng/ml; R&D Systems), IGF-1 (500 ng/ml; R&D Systems)]. The growth factors were added to the same spent medium that the cells were grown in and reapplied to the same cells to minimize false positive effects that could occur from addition of fresh medium. For STAT5 phosphorylation studies, immunoblots were probed with anti-pSTAT5A&B (05-495; Upstate, Lake Placid, NY) and total STAT5 with anti-STAT5 (9310; Cell Signaling Technology; Danvers, MA). AKT phosphorylation was detected with anti-pAKT (9271L; Cell Signaling Technology and total amounts with anti-AKT (9272; Cell Signaling Technology).

In flow cytometry signaling experiments, NCI-H838 cells,were starved as described above,vehicle (rHuEpo formulation buffer) or rHuEpo were added for 5 and 30 minutes then cells were fixed and processed as described previously [10]. Cells were then stained for 1 hour at room temperature with fluorochrome-conjugated antibodies that are specific for the phosphorylated forms of AKT (alexa fluor 647; Cell Signaling Technology), serine phosphorylated STAT3 (alexa fluor 488; BD Biosciences; San Jose, CA), STAT5 (alexa fluor 488; BD Biosciences) and S6 Ribosomal Protein (S6RP; alexa fluor 488; Cell Signaling Technology), then run on a FACS instrument (LSRII, BD Biosciences) to detect binding of antibodies. Data was analyzed using FACSDiva software (BD Biosciences). Results are reported as fold change in antibody binding (Mean Fluorescence Intensity (MFI) compared to vehicle addition alone.

## Results

### Specificity of Anti-EpoR Antibodies

We wished to compare EpoR protein expression in normal and tumor tissues using specific and sensitive anti-EpoR antibodies. However, we and others have reported that many antibodies used to examine EpoR protein expression were nonspecific [2], [6]–[8]. Thus experiments with those antibodies would not be helpful. Some antibodies, eg ab10653 showed some specificity to EpoR by western but the sensitivity was poor limiting its use [23]. Another monoclonal antibody (Mab307) showed specificity according to flow cytometry with intact cells but it recognized a conformational epitope and signal to EpoR was lost with denatured EpoR as is present in SDS-gels or formalin-fixed tissues, indicating it was not useful for Westerns or IHC [7]. Thus at the time of this writing there were no commercially available antibodies that would be useful for detection of EpoR in human tissue samples by western or immunohistochemistry.

Recently a novel rabbit anti-EpoR monoclonal antibody (A82) was described that could specifically detect low levels of human EpoR by western and flow cytometry offering potential in western experiments with human tissues [23], [24]. However, the absence of validated human EpoR negative control tissue types made it difficult to develop appropriate IHC methodologies with this and other antibodies, important in detecting false-positive and negative staining. One validation strategy would be to use murine tissues for which EpoR negative controls are available, e.g. EpoR knockout mice. However, A82 does not detect murine EpoR [24] and there are currently no other anti-murine EpoR antibody reagents available that have been properly validated for detecting EpoR by IHC.

To overcome these A82 difficulties, a human EpoR knockin (KI) mouse was employed where the human *EPOR* replaced the mouse *EPOR* coding sequences while retaining the mouse *EPOR* gene regulatory regions allowing for detection of human EpoR in a mouse. WT mouse tissues containing the murine *EPOR* gene from the same genetic background could serve as negative controls. Thus the same tissue types from KI and WT could be compared to assess antibody specificity. Spleens from KI and WT mice administered rMsEpo were previously shown to be suitable EpoR positive and negative controls respectively according to westerns with A82 [24]. In particular, spleens from rMsEpo treated mice have elevated EpoR expression because EpoR-expressing erythroid progenitor cells in spleen tissue increase significantly with rMsEpo [24].

**Figure 1:**
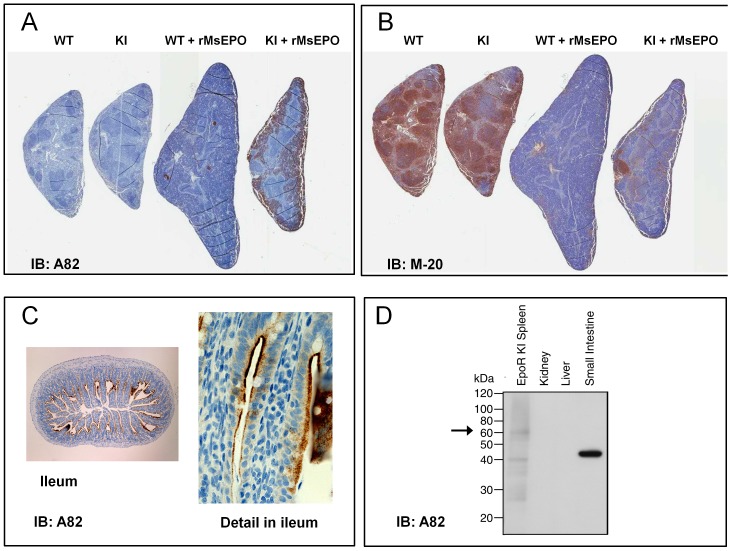
Binding of Anti-EpoR antibodies to spleens from WT or human EpoR KI mice. Spleens from untreated or rMsEpo-treated mice were sectioned and stained with anti-EpoR antibodies as described in Methods. Note the enlargement in spleen with rMsEpo addition due to enhanced erythropoiesis. (A) Staining with anti-EpoR monoclonal antibody A82. (B) The same sections as in A were stained with polyclonal antibody M-20. (C) Staining of mouse WT small intestine (ileum) with A82. Note the staining of the material in secretory granules and just under the surface. (D) A82 Western blot with mouse tissues. Note the ∼ 45 kDa protein non-EpoR protein in small intestine.

To assess the specificity of the A82 antibody by IHC and to determine specificity of other antibodies by IHC, spleen sections from untreated and rMsEpo-treated mice were prepared. In response to rMsEpo treatment, spleens from both WT and KI mice showed histological evidence of increased erythropoiesis in the red pulp (Figure 1A). The A82 antibody demonstrated a concomitant increase in staining of KI erythropoietic tissue consistent with elevated HuEpoR. As expected, there was no rMsEpo-mediated increase in A82 staining in WT erythropoietic tissue, although some spots of off-target staining of nonerythroid cells were observed in WT mice treated with rMsEpo.

Most WT mouse tissues, including WT mouse spleen with and without rMsEpo, showed essentially no binding of the A82 antibody (data not shown). However, there was some infrequent cross-reactivity in a limited panel of normal WT mouse and human tissues; primarily acellular components in some tissue sections. As one example, there was cross-reactivity with WT mouse ileum (Figure 1C). Western blots with A82 of WT tissues were performed with mouse small intestine and a band was detected but it was of a different size (∼45 kDa) compared to that observed with EpoR KI spleen (∼59 kDa for full length EpoR; Figure 1D) suggesting false-positive staining due to a non-EpoR cross-reacting protein. Given the occasional false-positive staining with some tissues, A82 is not suitable for IHC without appropriate positive and negative controls and follow-up with other methodologies to demonstrate the staining is specific to EpoR. Such follow-up should include, at a minimum, a western blot to ensure that the tissue contains EpoR proteins of the correct size. However A82 may show utility in cross-validation IHC experiments with other anti-EpoR antibodies should they be developed.

Another anti-EpoR antibody (M-20) was used to examine EpoR expression by Western/IHC in tumors and cell lines by multiple groups with positive results reported [9], [22], [30]–[34]. In order to determine if M-20 could specifically detect human or mouse EpoR in tissues by IHC, WT and KI mouse spleen tissues were tested. To compare staining patterns obtained with M-20 to that of A82, IHC with the 2 antibodies was performed with the same tissue blocks. As shown in Figure 1B there was strong staining by M-20 of WT mouse spleen that was similar to that of the KI mouse spleen from untreated mice. In this experiment, M-20 stained the T and B cell rich white pulp more strongly than the red pulp that is the main site of splenic erythropoiesis, suggesting cross-reactivity with something other than EpoR. Surprisingly, and in contrast to A82, the staining in spleens with M20 decreased with both WT and KI mice treated with rMsEpo. This is not consistent with the histological increase in erythropoiesis, the A82 IHC data on the same tissue blocks (Figure 1A) or with the increase in EpoR expression in spleen confirmed by westerns with A82 [24]. This suggests that M-20 can show false-positive staining by IHC and it should not be used for that application. We did not further explore M-20 for IHC experiments.

**Figure 2:**
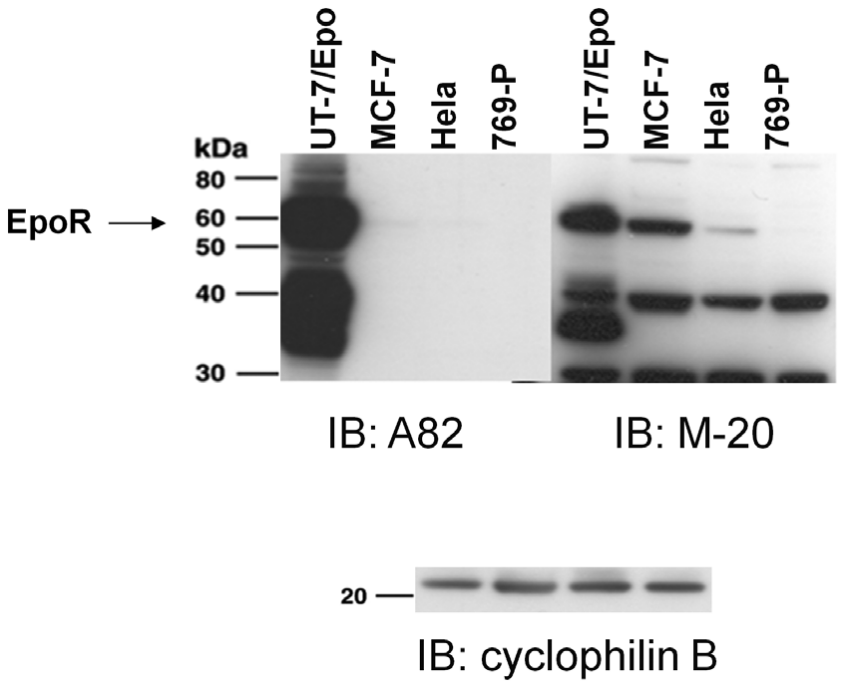
Specificity to EpoR of anti-EpoR antibodies A82 and M-20. EpoR protein expression analysis was performed by immunoblotting (IB) with anti-EpoR antibodies A82 or M-20 (lot E2004). Three identical blots using the same lysates were processed then probed with the indicated antibodies at the same time. The EpoR positive controls were a Cos-cell lysate overexpressing a FLAG-tagged version of Human EpoR [6] and UT-7/Epo cells. Negative controls were 769-P cells and COS cells transfected with an empty vector. The position of the 59 kDa full-length EpoR protein is indicated by the arrow. The positions of molecular weight markers are shown. The lower portion of the blot was also probed with anti-cyclophilin as a loading control.

To determine if M-20 could specifically detect EpoR by western and to better understand the conflicting western data between M-20 [9], [34] and A82 [10], multiple lots of M-20 were obtained, EpoR positive and negative control cell lysates were prepared and westerns were performed. Lysates of the breast cancer cell line MCF-7 were specifically included in these experiments because a band similar in size to EpoR was reportedly detected by M-20 [6], [9]but not A82 [10]in those cells. As shown in Figure 2, A82 detected full length EpoR (59 kDa) and smaller EpoR fragments in the positive control cells (UT-7/Epo) but not in MCF-7, Hela or negative control 769-P cells. Similar to A82, M-20 (lot E2004) detected a 59 kDa protein and smaller proteins in positive control UT-7/Epo cells and not in negative control 769-P cell lysates. However, additional smaller bands were observed with EpoR negative control 769-P cells indicating these were not EpoR proteins. In contrast to A82, there was a 59 kDa band detected by M-20 with MCF-7 cells and also with Hela cells but at lower level compared to MCF-7 cells. Similar results were observed with 2 other lots of M-20 (lots D232, G2103) while a 3^rd^ lot (K0910) showed only background staining with all lysates (data not shown). This positive M-20 data with MCF-7 is in concordance with that reported by others [6], [9], [32]. However this positive M-20 western data with MCF-7 and Hela cells is in contrast to that reported with 2 other anti-EpoR antibodies, ab10653 (Abcam Inc) and a rabbit polyclonal raised to murine EpoR with no 59 kDa proteins detected [23]. It is also not consistent with the low levels of EpoR transcripts in MCF-7 and Hela cells [5], [10], nor the undetectable cell-surface EpoR according to binding studies with radiolabeled rHuEpo. [5].

**Figure 3:**
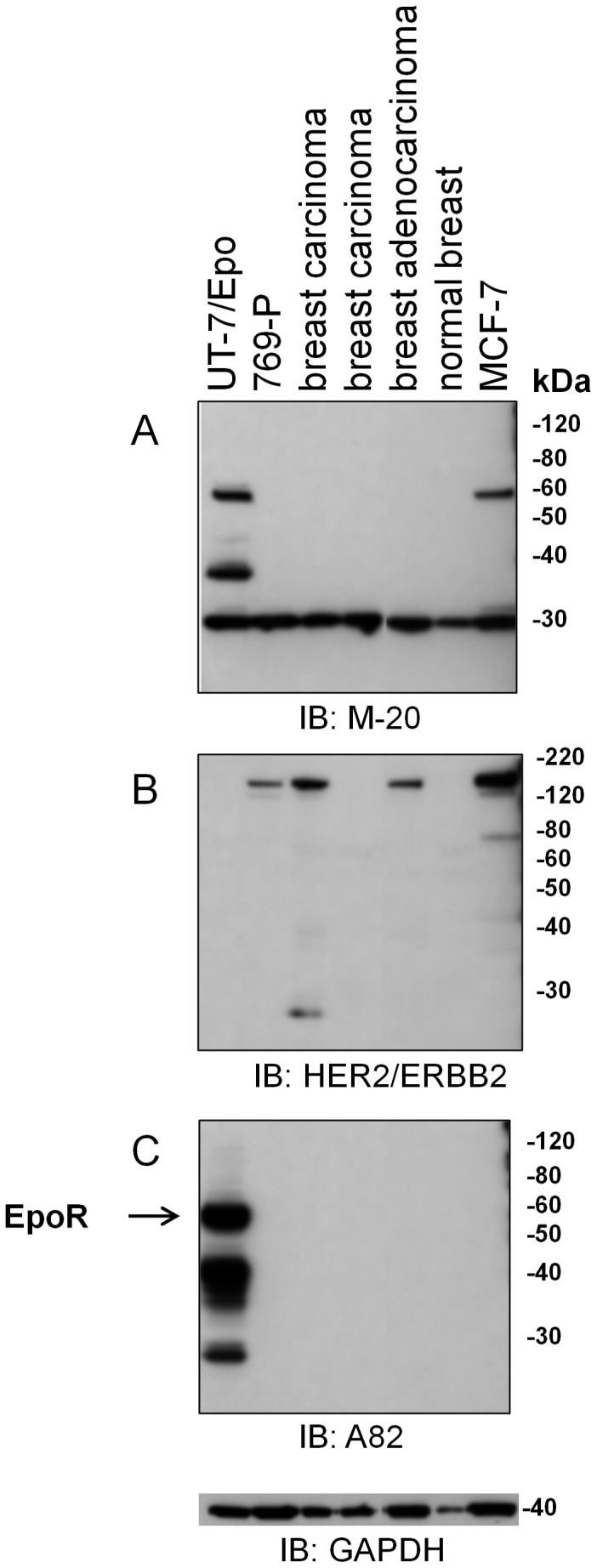
No EpoR detected by A82 or M-20 in normal and cancerous human breast tissues (Her2^+^ and Her2^−^) but M-20 detected a 59 kDa band in MCF-7 Cells. EpoR protein expression analysis was performed by immunoblotting (IB) with A82 and M-20. Four identical immunoblots with the same master mix were performed at the same time and probed with different antibodies under the same conditions. Lysates included tissue samples from 3 different human tumor biopsies, a normal breast sample or MCF-7 cells. The positive control was UT-7/Epo cells and the negative control was 769-P cells. The positions of molecular weight markers are shown. (A) Anti-EpoR monoclonal antibody M-20 (lot E2004). (B) Anti-Her2 antibodies. (C) Anti-EpoR antibody A82. A blot with anti-GAPDH antibodies served as a loading control.

In contrast to these MCF-7 results with M-20, EpoR was not detected by western with M-20 in either normal breast or tumor tissue (Figure 3A). These tumor samples included both Her2 positive and negative breast cancer tissues (Figure 3B). This lack of staining of breast cancer tissue by western with M-20 contrasts with positive IHC data with M-20 on breast cancer tissues reported previously [9], [34]. In this western experiment, M-20 also detected a band that migrated at approximately 25 kDa in every sample including the EpoR negative control 769-P sample indicating that this was not an EpoR protein fragment. This binding to the 25 kDa protein may at least partially explain the false-positive IHC staining in untreated mouse and human spleen tissue (Figure 1B) and the M-20 staining observed elsewhere with breast cancer tissues [9], [34].

**Figure 4:**
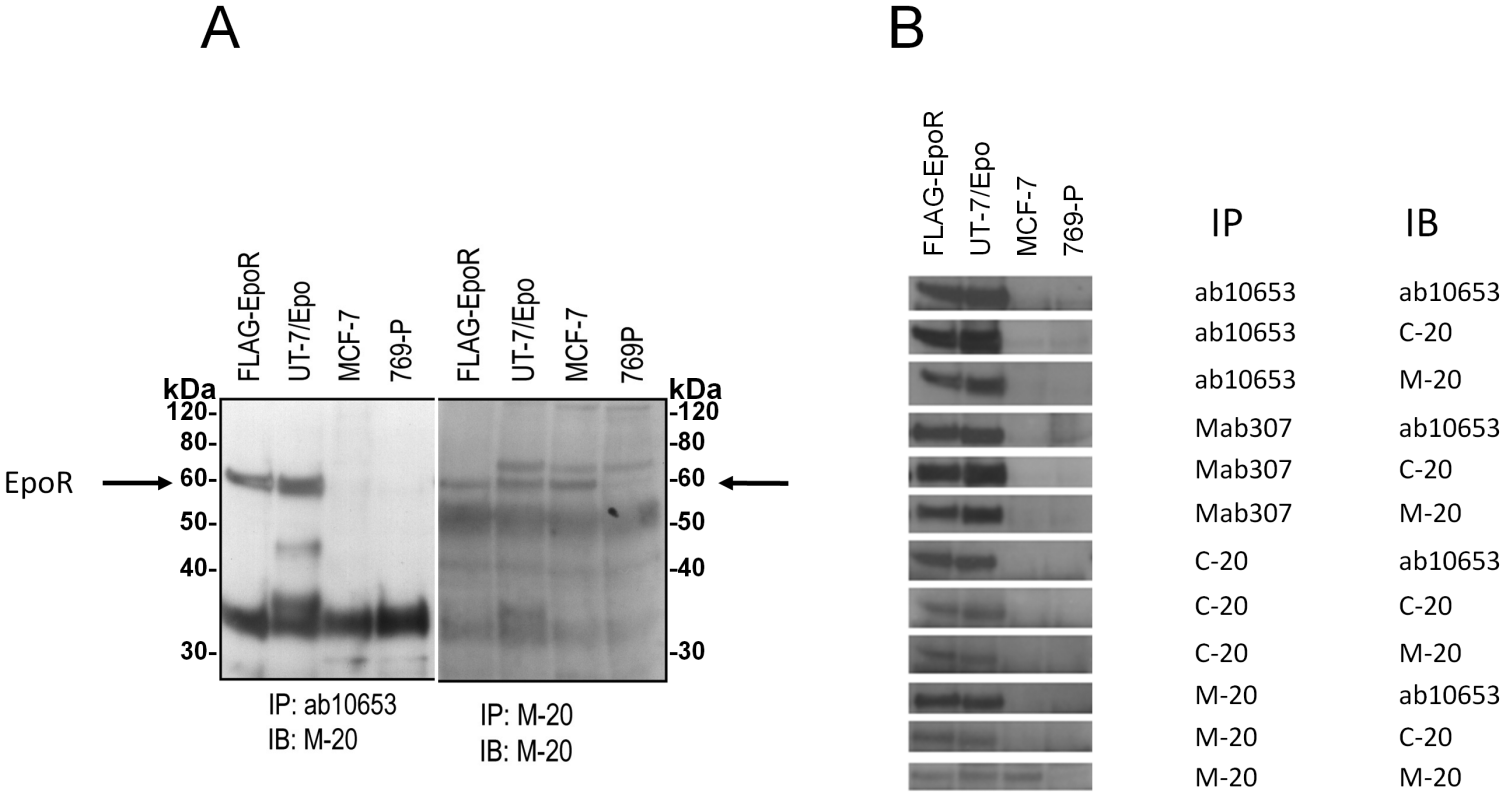
The 59 kDa protein detected by M-20 In MCF-7 cells is not bound by other anti-EpoR antibodies. The indicated lysates were immunoprecipitated (IP) then the immunoblotted (IB) with the indicated antibodies: ab10653 (abcam Inc), Mab307 (R&D systems), C-20 & M-20 (Santa Cruz Inc) or A-82 (Amgen Inc). COS cell lysates expressing a FLAG-tagged version of EpoR (FLAG-EpoR) [6] and UT-7/Epo cells served as EpoR positive controls. 769-P cells served as the EpoR negative control. (A) Westerns were immunoprecipitated (IP) with ab10653 or M-20 followed by immunoblotting (IB) with M-20. The position of full-length 59 kDa EpoR in positive controls is indicated by the arrow. Positions of molecular weight markers (kDa) are shown. Bands detected in 769-P lysates are non-EpoR cross-reacting proteins and include antibody chains that were not removed completely or protein G that leached from beads. Note the detection of a 59 kDa band with MCF-7 cells with the M:20/M:20 combination but not with the ab10653/IB:M-20 combination. (B) IP:IB combinations with the indicated antibodies were subjected to western analysis. The western slice containing the 59 kDa EpoR band from each combination is shown. Note the 59 kDa bands detected in EpoR positive controls but not 769-P cells. Only the M-20:M-20 combination detected a 59 kDa band in MCF-7 cells.

To further investigate the possibility that the 59 kDa protein detected by M-20 in MCF-7 was not EpoR, an immunoprecipitation:immunoblotting strategy (IP:IB) was employed with combinations of anti-EpoR antibodies. As shown in Figure 4A, when ab10653 was used to immunoprecipitate cell lysates, the subjected to SDS-PAGE and the blot then probed with M-20, a 59 kDa protein was detected in positive controls (FLAG-EpoR and UT-7/Epo) but not in negative control (769-P) or in MCF-7 cell lysates. There was a smaller protein detected in MCF-7 cells with this protocol but it was also seen in EpoR negative 769-P cells indicating it was not EpoR. In contrast to these results, a 59 kDa protein was detected with MCF-7 cell lysates when the lysate was first immunoprecipitated with M-20 and the blot was probed with M-20. To extend these studies, additional combinations of antibodies were used in IP:IB studies (Figure 4B). With all the other combinations of antibodies including some where M-20 was used for either to IP or IB, a 59 kDa protein was detected in the positive controls but no 59 kDa band was detected with the negative control (769-P) nor MCF-7 lysates. Taken together, these data suggest that the 59 kDa protein detected with MCF-7 by M-20 is likely a non-EpoR protein. That M-20 detected a protein the same size as EpoR highlights the limitation of similarity of size as a confirmatory result and further highlights the importance of additional controls to demonstrate antibody specificity. Because we would not be able to determine if the bands detected were EpoR or a similarly sized non-EpoR protein we did not examine EpoR expression further with M-20,

### Expression Profiling of EpoR in Human Normal and Tumor Tissues with A82

**Figure 5:**
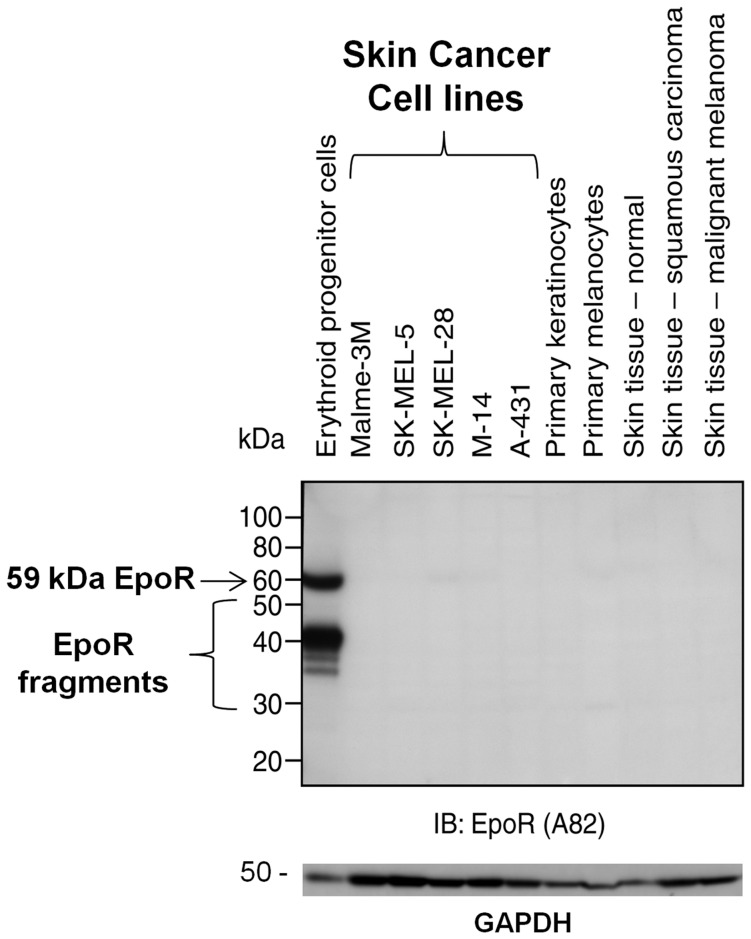
Anti-EpoR westerns with A82 on normal and tumor cell lines and tumor tissues. EpoR protein expression analysis was performed by immunoblotting (IB) with A82. Samples were processed and subjected to western using the anti-EpoR monoclonal antibody A82. With long exposures this antibody can specifically detect EpoR protein in cell lysates at levels as low as 100 fg (<20 molecules/cell) [23]. The positive control was erythroid progenitor cells. Positive control erythroid cells were prepared by culturing human CD34^+^ cells for 4–6 days in media that supported differentiation into Epo-responsive erythroid cells with physiologically relevant levels of EpoR^5^. The position of the 59 kDa full-length EpoR protein is indicated by the arrow. The proteins smaller than 59 kDa have been shown previously to contain EpoR sequences and are EpoR fragments [23]. Western with the indicated human normal and tumor tissue biopsies, primary cells and skin cell lines is shown. The blots were stripped and reprobed with anti-GAPDH antibodies to serve as a loading control. Positions of molecular weight markers are shown.

Given that A82 can specifically detect low levels of human EpoR by western analysis, it was used to determine if EpoR could be detected in human tumor biopsies. In previous experiments, skin tissue from humans and rodents had the lowest levels of EpoR transcripts of any tissue examined [2]. To explore the possibility that skin tissue may be a potential EpoR negative control for western and IHC, westerns with skin tissues and skin cell lines were performed. In this experiment, an EpoR band was readily detected by A82 in positive control erythroid progenitor lysates and not in negative control 769-P lysates and no EpoR was detected in any of the normal skin samples or skin tumor cell lines (Figure 5). Tissue from a skin carcinoma and a malignant melanoma was also included with no EpoR detected. The limit of detection with this particular protocol is conservatively estimated at approximately 100 EpoR dimers per cell [24], substantially below that of erythroid progenitor cells; ∼ 10,000 EpoR dimers/cell [10]. It is important to note that western blots detect total EpoR (surface and intracellular) but <10% of total EpoR protein finds its way to the cell surface [10], [35], [36]. This result suggests high level EpoR expression is not a universal property of skin carcinoma or melanoma and further indicates the value of skin as an EpoR negative control.

**Figure 6:**
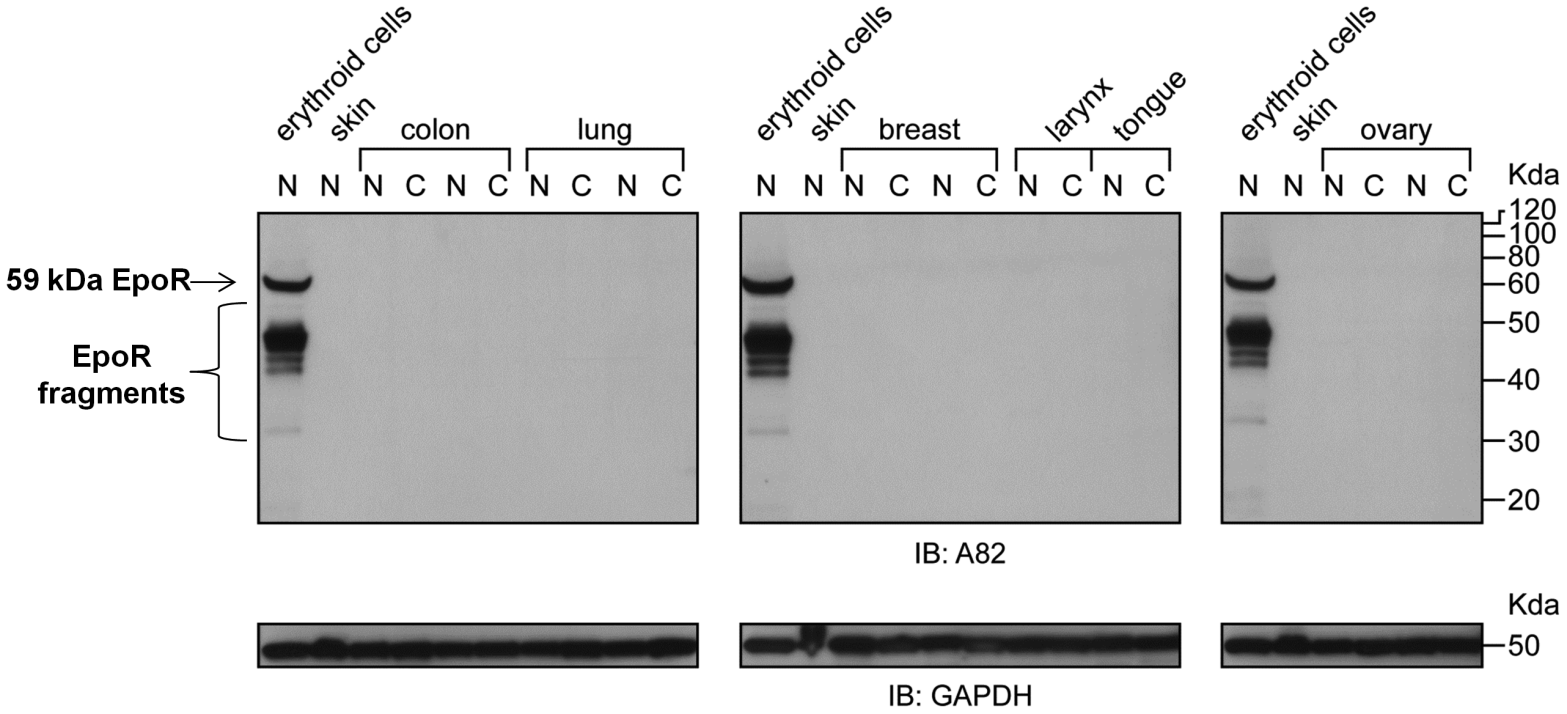
Anti-EpoR westerns with A82 on normal and tumor tissues. EpoR protein expression analysis was performed by immunoblotting (IB) with A82. The positive control was erythroid progenitor cells prepared as described in Figure 5. The position of the 59 kDa full-length EpoR protein is indicated by the arrow. Tissue biopsies from different human subjects with the indicated cancer types are shown. Samples were from cancerous regions (C), or adjacent regions containing normal cells (N) from the same patient. In this experiment skin tissue served as the negative control. The blots were stripped and reprobed with anti-GAPDH antibodies to serve as a loading control. Positions of molecular weight markers are shown.

Using skin as a negative control to detect false-positives, and in concordance with previous tissue western results [24], no EpoR protein was detected in normal tissues from colon, lung, breast, larynx, tongue or ovary (Figure 6) and there was no EpoR detected in the corresponding, matched tumor tissues. Taken together these results suggest that high level EpoR protein expression is not a general characteristic of tumors. However, with this experiment we cannot conclude that all possible tumors are EpoR negative.

### Expression of EpoR in Breast and Lung Cancer Cell Lines

**Figure 7:**
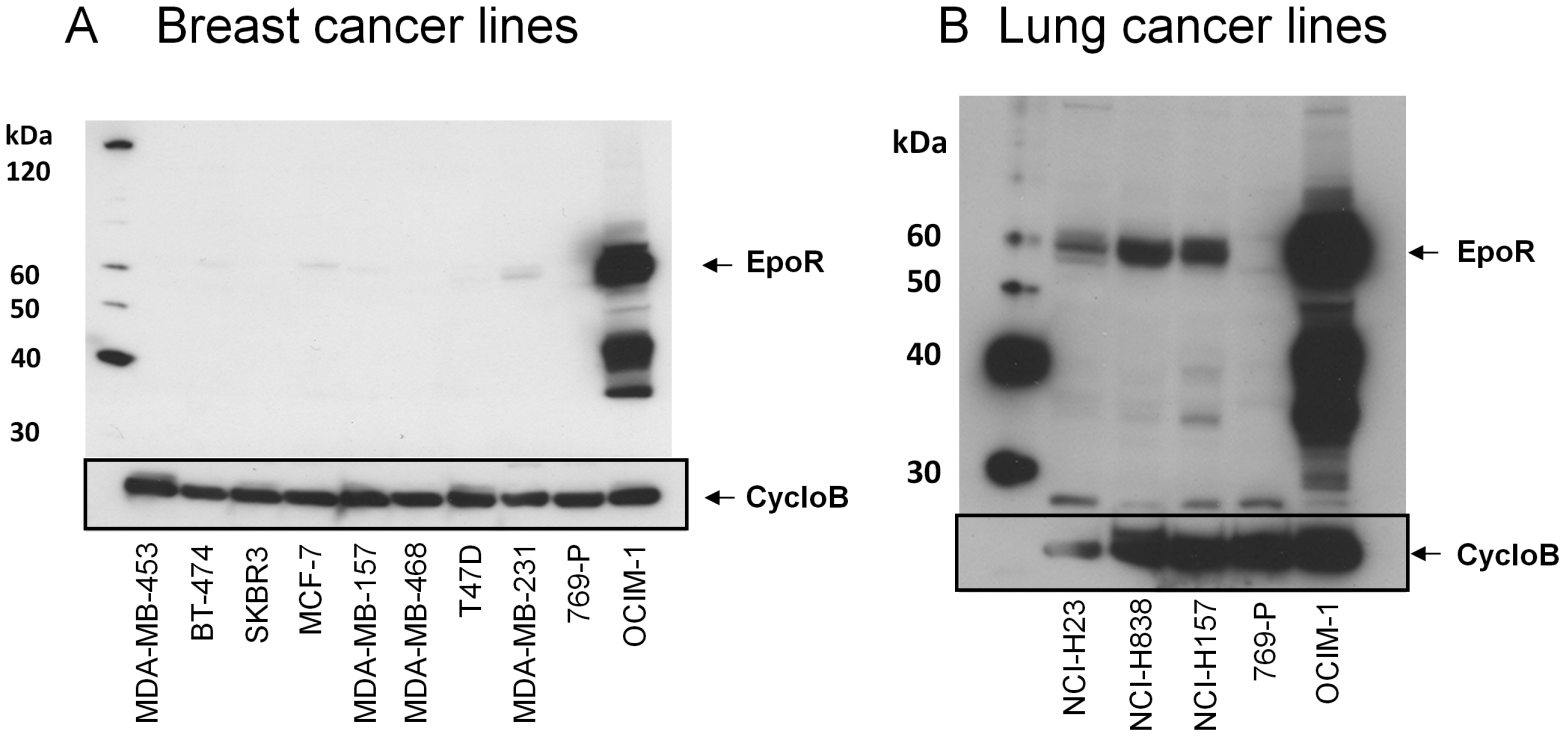
EpoR protein expression in breast and lung cancer cell lines. Breast and lung cancer cell lines were grown and subjected to western blotting with A82. Positions of molecular weight markers are shown. Full-length EpoR is indicated by the arrow. 769-P and OCIM-1 served as negative and positive controls for EpoR respectively. Blots were stripped and reprobed with anti-cyclophilin B to control for protein loading. (A) Breast cancer cell lines. (B) Three of the lung cancer lines that had detectable levels of EpoR.

In contrast to the western data with the tumor tissues above, in an earlier study, EpoR was detected in ∼ 10% of 60 different tumor cell lines derived from numerous tissue sources, but levels were still over 20-fold below that of positive controls [10]. However the lines with the higher levels of EpoR, had no detectable EpoR according to binding of [^125^I]-rHuEpo on the cell surface and they did not respond to rHuEpo. In contrast others have suggested that breast [9], [34] and lung tumor [22] cell lines expressed EpoR mRNA and protein and responded to rHuEpo. In our previous study 11 breast cancer lines and 20 lung cancer lines were examined with low/no EpoR detected in most cell lines [10]. To extend these studies, additional breast lines as well as the lung line NCI-H838 were examined by western with A82. OCIM-1 was included as a positive control. OCIM-1 cells express ∼ 10,000 EpoR dimers per cell which is comparable to that observed in erythroid progenitor cells [23]. EpoR was readily detected by western with A82 in OCIM-1 cells with no bands detected in 769-P cells. Similar to 769-P, most breast lines expressed undetectable EpoR but with very long exposures a full-length EpoR protein band was detected in several of the lines (Figure 7A). The highest levels of 59 kDa EpoR observed in these lines were <100 dimers/cell according to semi-quantitative immunoblotting estimates [10], 100-fold lower than that observed in positive control OCIM-1. Lung cancer cell lines found to have the higher levels of EpoR were included in a western with NCI-H838 (Figure 7B). NCI-H838 had EpoR protein that was ∼20% that of positive control erythroid cells and was comparable to the highest levels observed in other lung lines such as NCI-H157 cells.

### Functional Assessment of Responsiveness of NCI-H838 Cells to ESAs

**Figure 8:**
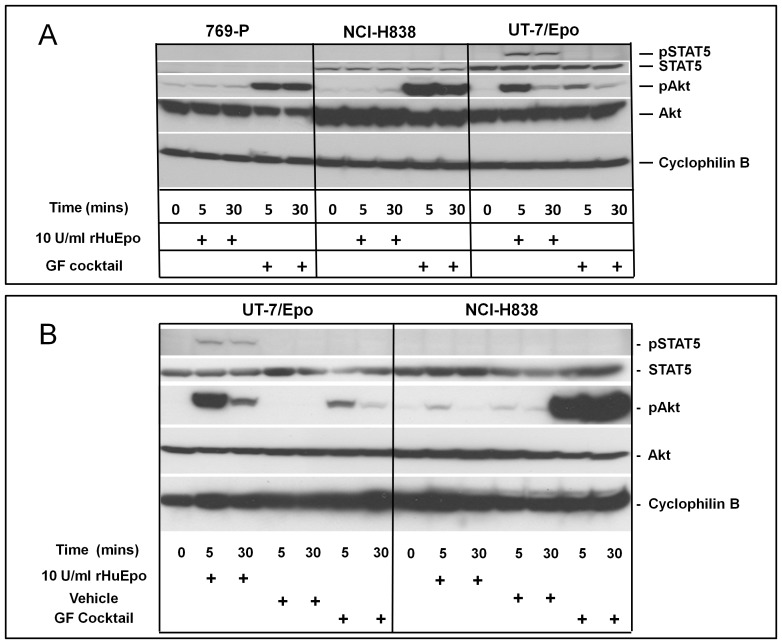
No effect of Epo on AKT or STAT5 phosphorylation with NCI-H838 lung carcinoma cell line. (A) NCI-H838 cells were serum and Epo-starved overnight in medium containing 0.1% serum then treated with the indicated growth factors for the indicated times in the same conditioned minimal medium (37°C) with added growth factors. Blots were probed with specific antibodies to the indicated total or phosphorylated molecules. Data shown using NCI-H838 cells obtained from ATCC. UT-7/Epo and 769-P cells served as positive and negative controls respectively. (B) Cells were starved in 0.0% FBS for 24 Hrs and then rHuEpo, Vehicle, or Growth Factor Cocktail Treatment (HGF, IGF-1 and EGF) was added to quiescent cells. Blots were probed with specific antibodies to the indicated total or phosphorylated molecules. NCI-H838 cells were obtained from T. Lappin University of Dublin, Ireland. UT-7/Epo served as a positive and control.

As noted above, the lung cancer cell line NCI-H838 was reported elsewhere to respond to rHuEpo with a robust increase in STAT5 phosphorylation [22]. These observations are notable because in a recent and exhaustive survey of the literature, this was the only nonerythroid tumor cell line that showed a significant increase in phosphorylation of STAT5 or JAK2 with rHuEpo [2]. Further, NCI-H838 cells had detectable EpoR according to A82 western (Figure 7B). The responsiveness of NCI-H838 to rHuEpo was therefore examined in detail with signaling assays. In these experiments, cells were serum starved to enhance the sensitivity of the assay and then cells were given rHuEpo. To avoid artifacts caused by media change, rHuEpo was diluted into conditioned medium from the same cell culture and rHuEpo containing medium was added back. Changes in phosphorylation were detected by western using specific anti-pAKT and pSTAT5 antibodies. Using this methodology with EpoR negative control 769-P cells, no changes were observed with either the experimental manipulation or with rHuEpo addition (Figure 8A). However increased phosphorylation of AKT and STAT5 was observed with addition of rHuEpo to Epo-responsive UT-7/Epo cells. UT-7/Epo cells also showed an increase in pAKT with growth factor addition (Figures 8A and 8B) with no effect on pAKT with the vehicle control (Figure 8B) indicating that those growth factors could specifically stimulate these cells. In agreement with a previous report [24] there was no effect on pSTAT5 with UT-7/Epo cells treated with the growth factor cocktail, likely because STAT5 is more specific to the Epo/EpoR axis and the growth factors in the cocktail (HGF, IGF-1 and EGF) signal through STAT1/3 [37], [38] and not STAT5. In contrast to UT-7/Epo and to that reported by Dunlop et al, [22], there was no observed effect of rHuEpo on levels of either pAKT or pSTAT5 with NCI-H838 cells.

One possible explanation for the negative results with rHuEpo here compared to that of Dunlop et al [22] are differences in the NCI-H838 lines used. Phenotypic and genotypic changes can occur with passages and adaptation/selection in different labs. To test this possibility, the same line [22] was obtained and the experiment was repeated with similar negative results (data not shown). So far, these experiments were performed in 0.1% serum but the previous positive data was observed with cells starved in serum-free medium. To determine if this difference could explain the disparate results, the experiment was further performed in serum free medium using the same protocol and anti-pSTAT5 antibody as was described [22] and again no effect of ESAs on pSTAT5 or pAKT was detected (Figure 8B).

**Figure 9:**
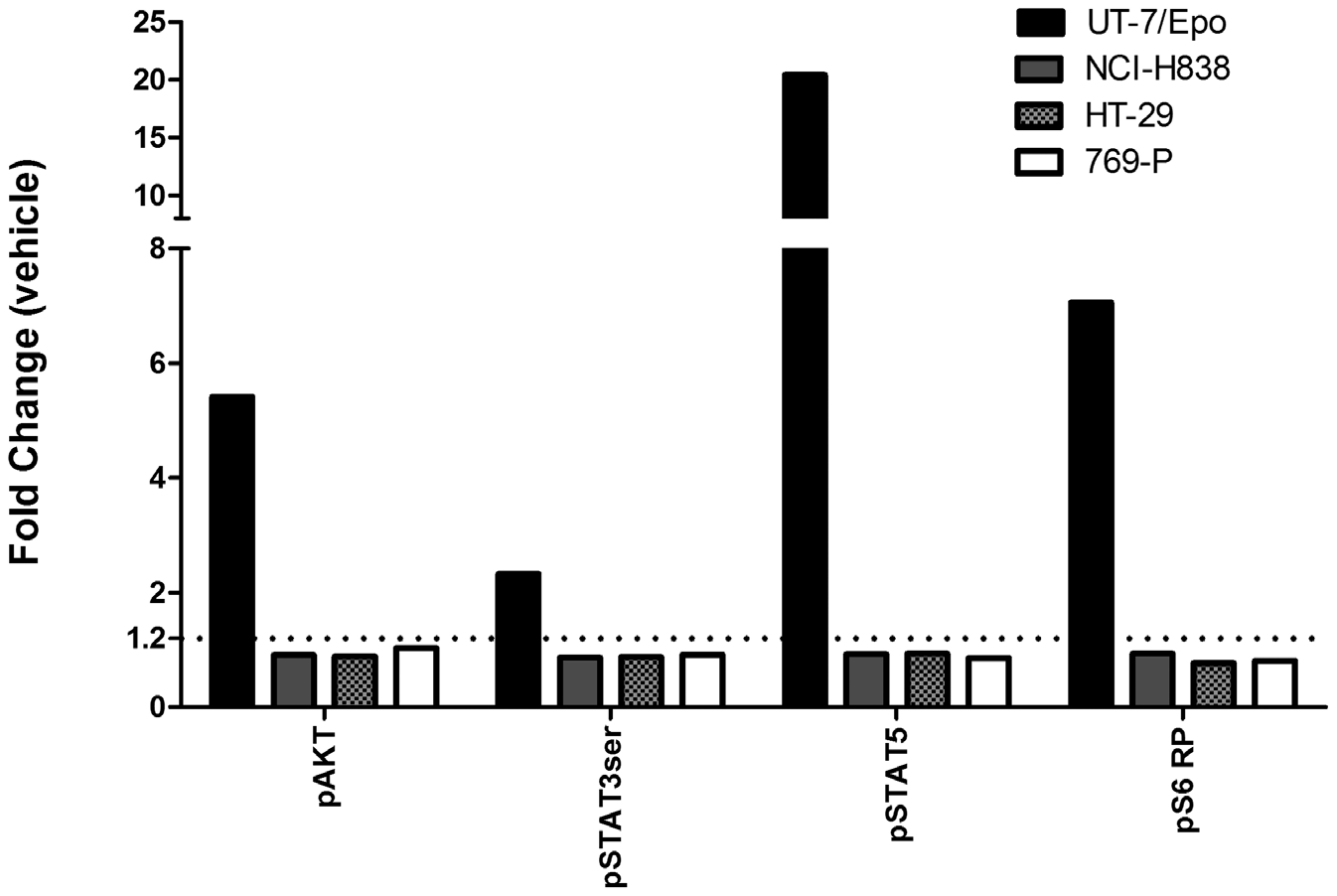
No effect of Epo on pAKT, pSTAT3, pS6RP or pSTAT5 with NCI-H838 lung carcinoma cell line by Phosflow. Cells were grown, treated for 30 minutes with the indicated growth factors and recovered. Fixed and permeabilized cells were washed and stained at room temperature with fluorochrome-conjugated antibodies specific for the phosphorylated (p) forms of STAT5, STAT3 and AKT, and S6 ribosomal (S6RP) proteins. Stained cells were run on a LSRII (FACS) instrument. For both vehicle and Epo treated samples 10,000 events were acquired during the FACS acquisition. Through gating and use of caspase 3 all events were from viable and intact cells. The data in the figure represents a single measurement for each cell line across all measured phospho-proteins. Results are reported as mean fluorescence intensity (fold change) in treated samples compared to vehicle. The dotted line shows the threshold level of staining that can be detected above background. This value represents the minimum fold change relative to vehicle required to accurately define a positive cytokine response signal in the Phosflow assay. The threshold fold change of 1.2 was experimentally validated previously through experiments where stimulated cells were titrated into negative control cells (data not shown). UT-7/Epo cells were the positive control and 769-P and HT-29 cells were negative controls.

We next performed the same experiment with our NCI-H838 cells but instead of westerns to detect phosphorylation of pAKT and pSTAT5, flow cytometry (Phos-flow) was performed with a larger panel of specific antibodies to phosphorylated molecules including pAKT, pSTAT3, pSTAT5 and pS6RP. This methodology assesses phosphorylation of specific molecules in single cells so potential stimulation of small numbers of cells in the population can be detected. In this experiment, 2-fold or greater increases in binding of antibodies to pAKT, pSTAT3, pSTAT5 and pS6RP were seen with rHuEpo addition with EpoR positive UT-7/Epo cells with no change with negative control HT-29 and 769-P cells (Figure 9). Notably pSTAT5 increased over 20-fold with rHuEpo in UT-7/Epo cells. However, similar to the western data, no increased phosphorylation above background was detected with any of the anti-phospho antibodies, including anti-pSTAT5, following rHuEpo addition to NCI-H838 cells.

## Discussion

In this paper we report that there was undetectable EpoR protein in human normal and tumor tissues from breast, lung, skin, colon and ovary. We also report that there was only no/low level (<100-fold lower than erythroid cells) expression of EpoR protein in a panel of breast tumor cell lines. While EpoR protein was detected in some lung tumor cell lines, the highest levels detected were <20% that of EpoR positive control cell types. This data is in concert with an earlier survey of tumor cell lines [10] 11 of which were breast cell lines and 20 were derived from lung cancers. It is also consistent with the no/low EpoR protein reported in normal human and mouse tissues examined with A82 western immunoblotting here and reported previously [24], [25].

In other studies, EpoR was detected in numerous tissues and cell lines according to sensitive RT-PCR methodologies but in accordance with our data, levels were similarly low in relationship to EpoR positive controls [2], [3], [8], [10], [25], [39]. Our data is also consistent with a lack of detectable binding of labeled rHuEpo to tumor cell lines [5], [10], [40]. Taken together these results suggest that high level expression of EpoR is not a general property of either normal or tumor tissues or tumor cell lines. However with this study we cannot eliminate the possibility that rare tumor types or rare cells within tumors express high levels of EpoR.

In contrast to these results, there are numerous reports that EpoR protein is expressed in tumors and tumor cell lines [2]–[4]. However, those data are based primarily on anti-EpoR antibodies with limited sensitivity and specificity to EpoR. [6], [41] [2], [6], [8], [41], [42]. One antibody examined in detail here (M-20) was thought to be specific to EpoR by IHC and western by others [9], [34], [43] and was also thought to be specific to EpoR in westerns according to our earlier report [6]. Described here we used mice bearing the human *EPOR* gene combined with negative control WT mice to show that by IHC M-20 could cross-react with an off-target binding element. This conclusion is supported by another report where there was staining by M-20 with an EpoR knockout embryo tissue section [6] showcasing the importance of tissue negative controls in demonstrating antibody specificity by IHC. Here and according to experiments with A82 vs. M-20 and with IP:IB methodologies it was further apparent that M-20 could provide false positive results by westerns. Notably M-20 detected a non-EpoR protein similar in size to full-length EpoR in some cell lines. These observations indicate that antibody validation experiments for westerns that are limited to positive and negative control tissues/cell types or even observation of a band at the correct size, cannot eliminate the possibility of false-positive results.

It is our experience that finding an antibody with absolute specificity is very rare but with appropriate controls and with some experimental protocols, useful data can be obtained with some antibodies. However additional confirmatory validation experiments are required. Notably A82 showed a high degree of specificity by western and by IHC with spleens from human *EPOR* bearing mice after rMsEpo addition and it did not show the same binding in negative controls, WT spleen with or without rMsEpo-treatment. According to this and other data [23] A82 appears to be one of the most sensitive and specific antibodies to EpoR currently available. However even A82 was shown to have some off-target cross-reactivity by IHC with some tissues, including negative control mouse spleen. In this particular case, we could conclude the staining was unlikely to be due to EpoR because the localization and staining pattern was not consistent with that observed with EpoR positive tissues such as spleen from rMsEpo treated human EpoR knock-in mice.

That monoclonal antibodies can cross-react with unrelated molecules is not new [44] and several reviews detail considerations when evaluating whether an antibody is suitable for IHC [45]–[49]. Many authors advocate the use of genetically modified mice where the human gene has been inserted or where a gene has been knocked out, to detect false-positive staining [45]–[49]. Furthermore as others have also found, specificity in some tissues does not guarantee specificity with all tissues which is one reason why large tissue panels are evaluated when assessing an antibody for clinical use [50]. However, the particular M-20 and A82 examples here highlight the importance of combinations of strategies to demonstrate antibody specificity and more importantly the importance of careful examination of the antibody validation strategies reported by others before personally using such antibodies. That M-20 detects a non-EpoR protein that is unfortunately the same size as EpoR further highlights the importance of additional methodologies to make definitive conclusions even for westerns.

Given the difficulties with anti-EpoR antibodies, support for the presence EpoR in tumors has come from functional experiments using rHuEpo on tumor cell lines but with conflicting results [2]. This is highlighted by our contrasting data with Dunlop et al with NCI-H838 cells [22]. Here despite detectable EpoR protein by western with A82, no detectable response of NCI-H838 to rHuEpo was observed. We have attempted to reconcile the discordant results but have been unable to find a definitive explanation. One possible difference was our reuse of cell conditioned medium when adding rHuEpo back to the cells to avoid artifacts associated with a fresh media change. However it is not clear if Dunlop et al also used this strategy. In any case, our results are in concert with other reports that tumor cell lines do not respond to ESAs [8], [10], [40], [43].

Others have reported positive effects with ESAs added to tumor cell lines [2]. However, despite careful examination of those publications, none were found that included negative controls, such as inclusion of true EpoR negative cell types, to detect false positive effects. Notably the positive signaling reports with tumor cell lines were primarily increases in phosphorylation of ERK or AKT [2]–[4], [9]. ERK and AKT are phosphorylated in response to activation of multiple growth factor receptor systems [17] making them prone to false-positive effects. Such false positive effects can be caused by changes in medium or addition of BSA (which can contain growth-promoting substances) [2]. Thus some of the positive data may be caused by subtle changes in stimulation or growth caused by the experimental manipulation. Indeed there are several reports of stimulation by ESAs of pERK or pAKT with an notable absence in the same study of increased phosphorylation of molecules more specific to EpoR activation such as pSTAT5 or pJAK2 [18], [20], [21], [32], [51]–[53]. In our experiments, media changes and experimental manipulations were minimized to reduce the likelihood of such false-positive effects.

The lack of a response to ESAs in the face of detectable EpoR protein expression has several explanations. The level of EpoR may be insufficient. In HEL cells, the magnitude of increase in phosphorylated JAK2 after rHuEpo addition, minimal in the parental cells, increased with overexpression of EpoR, demonstrating that level EpoR can affect the magnitude of a signaling response [54]. Increasing levels of EpoR in growth factor dependent cell lines caused them to become demonstrably Epo responsive [54]–[60] and the amount of EpoR in cell lines correlated with responsiveness to an Epo mimetic peptide [61]. The effect of EpoR level has also been shown to affect response in vivo. Mice that were haplo-insufficient (EpoR +/− mice) had reduced hematocrit and reduced responsiveness of their CFU-E to Epo compared to normal mice [62]. This can have physiological consequences and is consistent with the known biology of Epo and erythropoiesis where EpoR levels increase in concert with acquired Epo responsiveness [56], [58], [63]–[67]. Thus, low level protein production and/or inefficient surface translocation of EpoR may be limiting factors for Epo-EpoR responses in tumor cells and cell lines.

Another possibility explaining the lack of response to ESAs in cells containing EpoR may be an absence of or defects in signaling networks. Consistent with this proposal, the erythroleukemia cell line OCIM-1 does not respond to Epo (signaling or proliferation/survival) despite detectable EpoR expression on the cell surface [24], [27]. This may be due to constitutive phosphorylation (activation) of pSTAT5 and pERK pathways making them growth factor independent. Forced overexpression of EpoR resulted in Epo dependence for growth in some growth factor-dependent murine progenitor cell lines (FDCP-1, 32D, BaF3) but not in others, such as mouse IL-2 dependent T-cell lines HT-2 and CTLL2 or EGF-dependent NIH3T3 cells [68]–[77].These observations may be at least partly explained by constitutive activation of pathways making them non-responsive to cytokine challenge [78]. However, multiple explanations may be in play.

## Acknowlegments
